# Heterogeneity in the level of dementia literacy among community doctors in China: A latent profile analysis

**DOI:** 10.7189/jogh.14.04161

**Published:** 2024-11-15

**Authors:** Shuxian Qiu, Mei Zhao, Haifeng Zhang, Tao Li, Weihong Kuang, Sha Liu, Yongan Sun, Mingwei Wang, Hengge Xie, Enyan Yu, Xin Yu, Huali Wang

**Affiliations:** 1 Dementia Care and Research Center, Peking University Institute of Mental Health (Sixth Hospital), Beijing, China; 2 Beijing Municipal Key Laboratory for the Translational Research on Diagnosis and Treatment of Dementia, Beijing, China; 3 National Health Commission Key Laboratory of Mental Health, National Clinical Research Center for Mental Disorders (Peking University), Beijing, China; 4 Beijing Haidian Psychological Rehabilitation Hospital, Haidian District, Beijing, China; 5 Mental Health Center, West China Hospital, Sichuan University, Chengdu, China; 6 Department of Psychiatry, First Hospital/First Clinical Medical College of Shanxi Medical University, Taiyuan, China; 7 Department of Neurology, Peking University First Hospital, Beijing, China; 8 The First Hospital of Hebei Medical University, Shijiazhuang, Hebei, China; 9 Department of Geriatric Neurology, the Chinese PLA General Hospital, Beijing, China; 10 Department of Psychological Medicine, Cancer Hospital of the University of Chinese Academy of Sciences, Zhejiang Cancer Hospital, Hangzhou, China

## Abstract

**Background:**

This study aimed to explore the heterogeneity of community doctors based on their knowledge of dementia and the potential factors associated with their dementia literacy.

**Methods:**

A total of 1288 community doctors completed the Alzheimer disease knowledge scale (ADKS) in a cross-sectional study conducted between December 2021 and January 2022. We used latent class analysis (LCA) to explore potential clusters based on responses to the ADKS. Multivariate multinomial logistic regression analysis was performed to evaluate the associations between potential risk factors and the knowledge of community doctors.

**Results:**

Community doctors were divided into four clusters according to their knowledge structure (Akaike information criterion (AIC) = 35672.83, Bayesian information criterion (BIC) = 36307.63, adjusted BIC (aBIC) = 35916.91, entropy = 0.814): the severity-focused subgroup (n = 269), the physical issues-focused group (n = 370), the knowledge uncertainty group (n = 191), and the general-focused group (n = 458). Age, education level, type of practice, and professional title were associated with the knowledge structure of Alzheimer’s disease (AD). In addition, the perception that patients seek care in community health centres for physical reasons and community doctors’ failure to manage patients with recently identified cognitive impairment were associated with the structures of the ADKS among community doctors (*P* < 0.05).

**Conclusions:**

There is heterogeneity in the level of AD knowledge among community doctors and their demographic characteristics, perceptions, and practices. Further efforts are needed to optimise the knowledge structure of dementia among community health care professionals.

Alzheimer’ disease (AD) is a neurodegenerative disorder characterised by progressive cognitive decline that affects memory, thinking, behaviour, and daily activities [1]. The number of persons living with dementia will increase dramatically, posing a significant burden on global public health [2]. In China, the number of people with dementia is also substantial, with an estimated 15 million individuals aged 60 and above affected, with a prevalence rate of 6.0%, including nearly one million patients with AD [3]. In response to the potential dementia care burden, more attention has been placed to increase early detection of dementia, primarily through referral from primary care settings. Therefore, addressing the attitude and capacity of primary care professionals, such as community doctors, is a hot topic for dementia care and prevention promotion.

Research indicates that there is a pervasive lack of understanding and awareness of dementia among health care professionals, which often results in the condition's stigmatisation and impedes both diagnosis and caregiving [4]. Additionally, primary health care doctors need more training and access to AD knowledge, resulting in difficulty in accurately assessing and managing AD patients [5]. To date, numerous studies have explored understanding of dementia and its associated factors, including differences in AD knowledge between caregivers and non-caregivers, between pharmacists and general practitioners, and between individuals from different ethnic backgrounds or regions [6–8]. Alacreu et al. found that, although general practitioners have obtained substantial knowledge of AD in terms of diagnosis, treatment, and symptomatology, they exhibited significant gaps in identifying risk factors [7]. Another study in India similarly found that the level of AD knowledge among health care workers was below average [9].

Additionally, studies have found that AD knowledge among medical and nursing students varies with different educational backgrounds [10–13]. This trend is also observed in China, where medical personnel exhibit an inadequate grasp of AD, irrespective of their practice setting. Research has indicated that general practitioners in primary health care institutions in Beijing have misconceptions about dementia and believe that dementia management should be the responsibility of specialists, resulting in an inadequate understanding of the disease [14]. Further research on the knowledge and attitudes of community health professionals concerning dementia has shown that, despite a positive attitude, they have a deficient overall understanding of the condition, underscoring the imperative for enhanced educational initiatives targeted at AD within the community health sector [15].

A substantial body of research has explored the determinants of dementia knowledge levels, encompassing factors such as gender, age, ethnicity, educational attainment, and clinical experience. Existing literature suggests that females exhibit a more profound comprehension of AD knowledge [16–23]. Nevertheless, the influence of age on AD knowledge levels has yielded inconsistent findings across studies [21,24–27]. The level of education has been confirmed as a pivotal factor associated with AD knowledge, with individuals possessing higher levels of education being more prone to having a robust understanding of AD [10,21,28]. In addition, several studies have examined the relationship between clinical experience and AD knowledge levels, revealing that clinical experience correlates with a more proactive approach to diagnosing and treating AD. However, these findings are also subject to influences such as the type of medical practice, duration of professional experience, or academic credentials [10,11,28]. No studies have yet specified the content of clinical practice in this context.

Previous research has demonstrated disparities in the levels of AD knowledge among different demographic groups, especially concerning different domains of dementia knowledge. For instance, staff members in Chinese community service centres demonstrate high accuracy in treatment, management, and life impact but need more understanding of symptoms and caregiving aspects [29]. Chinese nursing and medical students exhibit significant differences in their knowledge of the life impact and symptoms [12]. Pharmacists and general practitioners score higher on diagnosis, treatment and management, and symptoms but lower on physical aspects of AD [7]. Caregivers outperform non-caregivers in symptoms, assessment and diagnosis, and disease progression [8]. These findings indicate the potential for heterogeneity in the comprehension of AD knowledge. The Alzheimer’ disease knowledge scale (ADKS) is one of the most commonly used instruments to measure the understanding of dementia in different populations. Previous studies have used item response theory procedures to explore the item characteristics of the ADKS. However, they only investigated the latent structure of ADKS items without explicitly identifying the knowledge structures of the sample population [8,30]. They could not reflect the heterogeneity of the studied population regarding their knowledge of AD.

Therefore, the primary objective of this study was to identify clusters of individuals from a nationally representative sample of community doctors in China and to determine the potential factors associated with the heterogeneity of dementia knowledge among community doctors.

## METHODS

### Study design

This study employed a cross-sectional design. Considering the inclusion of community doctors from all provinces nationwide in our study population, an online survey methodology was implemented. Using convenience sampling, we distributed SurveyStar platform links for a research questionnaire between 1 December 2021 and 7 January 2022.

Before initiating the survey, we drafted the questionnaire based on studying existing literature and previous community practice experience. The questionnaire was finalised through a two-round modified Delphi consultation, and then input to the SurveyStar platform. The web link of the online questionnaire was disseminated to participants via professional social media, i.e. WeChat group communication.

During the survey, participants were briefed on the survey questionnaire to ensure transparency while safeguarding the integrity of their responses. Mandatory fields were established for the questionnaire to guarantee the completeness of the collected data. Additionally, we have imposed restrictions to ensure that each participant can only complete the survey once, thus precluding the possibility of duplicate submissions. We then monitored the data collection daily. For provinces with lower engagement, we encouraged the academic committee members of those provinces to disseminate the questionnaire further to ensure adequate participation from each province. Ultimately, all returned questionnaires were inspected and uniformly encoded.

### Participants

The study enrolled 1288 community health care workers from several provinces in China. The study participants were recruited through the specialist-community collaborative network among members of the associations and academic groups on Alzheimer disease and related disorders, including Alzheimer’s Disease Chinese (ADC), Academy of Dementia and Cognitive Disorders in China, Chinese Society of Geriatric Psychiatry, Family Doctors Branch of Chinese Aging Well Association, and the provincial entities of these associations. Inclusion criteria are as follows:

1) individuals who have been employed at a community health service centre for a minimum of one year

2) individuals should be engaged in frontline clinical or public health medical services.

Exclusion criteria are as follows:

1) immediate family members of employees in AD diagnostic technology or drug companies

2) individuals who do not participate in frontline medical work within the past year and are not expected to participate in the coming year

3) individuals who do not complete the survey questionnaire.

Ethical approval for the study was obtained through an expedited review by the Institutional Review Board of Peking University Sixth Hospital. Before the commencement of the survey, all participants provided online consent. All collected data were anonymised and used solely for research purposes, thereby strictly protecting the privacy rights of the survey participants. Additionally, at the end of each section of the questionnaire, a question asked whether the participant consented to proceed to the next section. Responding ‘yes’ allowed them to continue to the next part, while answering ‘no’ terminated the survey. Such procedures thoroughly ensured the right to informed consent.

### Instruments

#### Alzheimer’s disease literacy

The Chinese version of the ADKS was used to assess Alzheimer’s disease literacy [31,32]. The study found that the Chinese version of the ADKS scale has a split-half reliability of 0.556, a Cronbach's α coefficient of 0.756, and a test-retest reliability of 0.816, demonstrating satisfactory psychometric properties regarding reliability and validity [33]. The ADKS comprises 30 statements and covers seven domains, including risk factors, assessment and diagnosis, symptoms, course of the disease, impact on life, caregiving, treatment and management. The 30 items consist exclusively of true-or-false questions, where each correct response scores 1 point and incorrect responses are not penalised, yielding a total score from 0 to 30 – a higher score indicating a more proficient grasp of AD knowledge.

#### Sociodemographic measures

All study participants responded to questions about their sociodemographic characteristics, including age, gender, educational attainment, professional title, the type of community health care services they were involved in, the duration of their occupational experiences as health care workers, and their practice in community health centres. The coding method for each variable is detailed in Table S1 in the **Online Supplementary Document**.

#### Healthcare practice

Three main questions asking community doctors about their clinical practice are as follows:

1) Please estimate the proportion of common reasons that elderly patients visit community doctors and seek advice. Three sub-questions were asked about the proportion of physical conditions, cognitive decline, and emotional and behavioural problems.

2) For elderly patients presenting with emotional and behavioural problems or physical conditions, is there a proactive approach to inquiring about changes in cognitive functions?

3) Please estimate the frequency of the four types of clinical management for elderly patients presenting with recent cognitive decline. Four sub-questions were asked about the frequency of taking no action, conducting cognitive screenings, referring to higher-level hospitals based on the outcomes of cognitive screenings, or making direct referrals to higher-level hospitals, respectively.

In this study, we recoded the original responses into dichotomy variables to facilitate the data analysis. The detailed variables coding methods are presented in Table S1 in the **Online Supplementary Document**.

### Study quality control

All questions were set to be required items. If any items were missed, the study participants could not submit their responses successfully. This approach ensured the completeness of the questionnaire.

### Statistical analysis

Potential latent analysis was conducted using Mplus version 8.2, assuming that a potential categorical variable can explain the relationship among a set of observation variables [34]. A constellation of statistical approaches encompassing the Akaike information criterion (AIC), the Bayesian information criterion (BIC), the adjusted Bayesian information criterion (aBIC), the Vuong-Lo-Mendell-Rubin likelihood ratio test (VLMR-LRT), the bootstrap likelihood ratio test (BLRT), and entropy, were employed to thoroughly evaluate and identify the most superior model [35,36].

After analysing models ranging from one to six clusters, we found that the four-cluster model achieved the smallest absolute BIC, with an entropy index of 0.814. Furthermore, the LMR and the BLRT reached significant levels in group four. Consequently, the four-cluster model was identified as optimal (Table S2 in the **Online Supplementary Document**).

We discerned distinct knowledge structures among community physicians and conducted χ^2^ tests for categorical variables to assess differences using *R* software (version 3.6.2, Auckland, New Zealand, 2019). The Kruskal-Wallis test was applied for continuous variables, followed by post hoc analysis (*P* < 0.05).

We utilised multivariate adjusted multinomial logistic regression analysis to evaluate the influence of sociodemographic attributes and clinical practices on AD knowledge structures while controlling for other variables. Statistical significance was set at bilateral *P* < 0.05 with 95% confidence intervals (CIs).

## RESULTS

### Sociodemographic characteristics and health care experiences of the study participants

A total of 1288 community health care workers actively participated in the study. Their average age was 38.53 years (SD = 9.16). The gender distribution comprised 30.2% males. Most participants had a high level of education and substantial health care experience, with 67.47% holding an undergraduate degree or above and 80.13% having more than five years of health care experience. Concerning the occupational type, 72.59% were general practitioners, 7.45% were psychiatrists, 6.06% were family doctors, and 13.9% were public health doctors, with more than 35% of community health care workers holding the position of consultant practitioners (**Table 1**).

**Table 1:** Sociodemographic and clinical practice characteristics of the total sample and the sample by the different subgroups

Variable	Participants (%)			SG 1* (n = 269)	SG 2† (n = 370)	SG 3‡ (n = 191)	SG 4§ (n = 458)	*P*-value	H/x^2^	SG 1 vs. SG 2	SG 1 vs. SG 3	SG 1 vs. SG 4	SG 2 vs. SG 3	SG 2 vs. SG 4	SG 3 vs. SG 4
**Age, x̄ ± SD**	38.53 ± 9.16			36.98 ± 8.72	38.42 ± 8.88	36.68 ± 10.32	40.29 ± 8.81	<0.001	37.131	−1.721	0.906	−4.704a	2.509	−3.196	−5.189
**Sex**								0.681	1.506						
Male	389 (30.20)			85 (31.60)	111 (30)	51 (26.7)	142 (31.00)								
Female	899 (69.80)			184 (68.4)	259 (70)	140 (73.3)	316 (69.00)								
**Education**								<0.001	82.331	6.809	22.336‖	40.810‖	28.500¶	22.795¶	88.599**
Technical secondary school	80 (6.21)			14 (5.20)	22 (5.95)	30 (15.71)	14 (3.06)								
Junior college	339 (26.32)			92 (34.20)	101 (27.30)	74 (38.74)	72 (15.72)								
Bachelor degree	784 (60.87)			139 (51.67)	225 (60.81)	82 (42.93)	338 (73.80)								
Master degree or above	85 (6.60)			24 (8.92)	22 (5.95)	5 (2.62)	34 (7.42)								
**Medical practitioner type**								<0.001	103.35	3.009	10.415	49.033	23.255¶	40.722¶	100.537**
General practitioner	935 (72.59)			182 (67.66)	272 (73.51)	102 (53.40)	379 (82.75)								
Psychiatrist	96 (7.45)			18 (6.69)	18 (4.86)	14 (7.33)	46 (10.04)								
Family doctor	78 (6.06)			19 (7.06)	25 (6.76)	22 (11.52)	12 (2.62)								
Public health doctor	179 (13.90)			50 (18.59)	55 (14.86)	53 (27.75)	21 (4.59)								
**Time working in clinical or public health**								<0.001	32.61	−0.827	1.153	−4.119‖	1.968	−3.579¶	−4.940**
<5 y	256 (19.88)			63 (23.42)	65 (17.57)	58 (30.73)	70 (15.28)								
5–9 y	328 (25.47)			71 (26.39)	115 (31.08)	49 (25.65)	93 (20.31)								
10–14 y	235 (18.25)			51 (18.96)	71 (19.19)	25 (13.09)	88 (19.21)								
>15 y	469 (36.41)			84 (31.23)	119 (32.16)	59 (30.89)	207 (45.20)								
**Time working in community health centre**								0.049	7.861	−1.214	0.836	−2.289	1.312	−1.124	−2.268
<5 y	396 (30.75)			86 (31.97)	105 (28.38)	68 (35.60)	137 (29.91)								
5–9 y	330 (25.62)			80 (29.745)	106 (28.65)	51 (26.70)	93 (20.31)								
10–14 y	202 (15.68)			40 (14.87)	60 (16.22)	22 (11.52)	80 (17.47)								
>15 y	360 (27.95)			63 (23.42)	99 (26.76)	50 (26.18)	148 (32.31)								
**Professional title**								<0.001	109.61	4.279	14.207‖	38.193‖	26.931¶	33.687¶	83.903**
Senior professional	47 (3.65)			12 (4.46)	10 (2.70)	4 (2.09)	21 (4.59)								
Deputy senior professional	189 (14.67)			29 (10.78)	41 (11.08)	13 (6.81)	106 (23.14)								
Intermediate professor	474 (36.80)			89 (33.08)	146 (39.46)	47 (24.61)	192 (41.92)								
Junior professor	463 (35.95)			112 (41.64)	144 (38.92)	90 (47.12)	117 (25.55)								
Others	115 (8.9)			27 (10.04)	29 (7.84)	37 (19.37)	22 (4.80)								
**Memory clinic in their area**								0.009	11.444	7.217‖	0.151	1.650	8.984¶	3.114	2.543
Yes	201 (15.61)			51 (18.96)	41 (11.08)	39 (20.42)	70 (15.28)								
No	1087 (84.39)			218 (81.04)	329 (88.92)	152 (79.58)	388 (84.72)								
**Memory clinic in their community health centre**								0.001	15.851	4.146	0.034	10.644‖	5.032	0.959	10.559**
Yes	74 (5.75)			24 (8.92)	17 (4.59)	18 (9.42)	15 (3.28)								
No	1214 (94.25)			245 (91.08)	353 (95.41)	173 (90.58)	443 (96.72)								
**The estimates of the proportion of reasons that elderly patients visit community doctors and seek advice**															
Physical conditions								<0.001	63.174	1.196	2.047	33.985‖	6.515	25.962¶	44.596**
*≥50%*	567 (44.02)			96 (35.69)	149 (40.27)	56 (29.32)	266 (58.08)								
*≤49%*	721 (55.98)			173 (64.31)	221 (59.73)	135 (70.68)	192 (41.92)								
Cognitive decline								0.589	1.922						
*≥50%*	246 (19.10)			55 (20.45)	70 (18.92)	30 (15.71)	91 (19.87)								
*≤49%*	1042 (80.90)			214 (79.55)	300 (81.08)	161 (84.29)	367 (80.13)								
Emotional and behavioural problems								0.732	1.286						
*≥50%*	191 (14.83)			43 (15.98)	52 (14.05)	32 (16.75)	64 (13.97)								
*≤49%*	1097 (85.17)			226 (84.01)	318 (85.95)	159 (83.25)	394 (86.03)								
Proactively inquiring about changes in cognitive functions for elderly patients presenting with emotional and behavioural problems or physical conditions								0.089	6.523						
*Usually*	773 (60.02)			172 (63.94)	230 (62.16)	117 (61.26)	254 (55.46)								
*Less often or none*	515 (39.98)			97 (36.06)	140 (37.84)	74 (38.74)	204 (44.54)								
**The estimates of the frequency of clinical management for elderly patients with recent cognitive decline**															
Taking no action								<0.001	24.26	2.618	0.014	18.889‖	2.022	7.695¶	14.269**
*Usually*	462 (35.87)			117 (43.49)	136 (36.76)	82 (42.93)	127 (27.73)								
*Less often or none*	826 (64.13)			152 (56.51)	234 (63.24)	109 (57.07)	331 (72.27)								
Conducting cognitive screenings								<0.001	19.201	3.680	0.284	11.840‖	5.624	2.169	13.533**
*Usually*	492 (38.20)			120 (44.61)	136 (36.76)	90 (47.12)	146 (31.88)								
*Less often or none*	796 (61.80)			149 (55.39)	234 (63.24)	101 (52.88)	312 (68.12)								
Referring to higher-level hospitals based on the outcomes of cognitive screening								0.382	3.060						
*Usually*	673 (52.25)			146 (54.28)	197 (53.24)	89 (46.60)	241 (52.62)								
*Less often or none*	615 (47.75)			123 (45.72)	173 (46.76)	102 (53.40)	217 (47.38)								
Making direct referrals to higher-level hospitals								0.078	6.819						
*Usually*	739 (57.38)			169 (62.83)	214 (57.84)	97 (50.79)	259 (56.55)								
*Less often or none*	549 (42.62)			100 (37.17)	156 (42.16)	94 (49.21)	199 (43.45)								

ADKS – Alzheimer’s disease knowledge scale, H – Kruskal-Wallis test, SD – standard deviation, SG – subgroup, x̄ – mean

*Severity-focused group.

†Physical issue-focused group.

‡Knowledge uncertainty group.

§General focused group.

‖*P* < 0.05 compared with SG 1.

¶*P* < 0.05 compared with SG 2.

**P < 0.05 compared with SG 3.

### Latent class analysis

**Figure 1:**
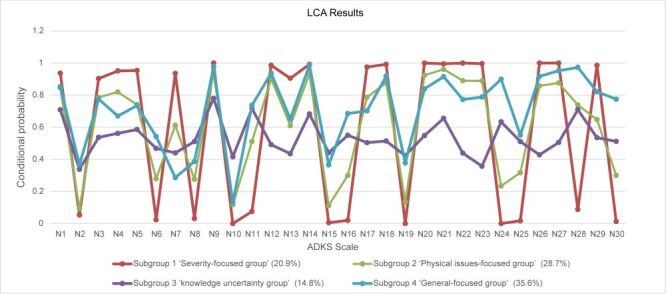
Potential subgroup conditional probability line chart of total ADKS scores of community doctors. N1-30: item 1-30 of the Alzheimer’s disease knowledge scale. AKDS – Alzheimer’s disease knowledge scale, LCA – latent class analysis.

According to the LCA models, the participants were divided into four subgroups according to their responses to the ADKS:

1) a subgroup that considered AD to be severe or damaging (severity-focused group, 20.9%)

2) a subgroup that concentrated on the physical disorder (physical issues-focused group, 28.7%)

3) a subgroup that did not show a specific preference for the ADKS items (knowledge uncertainty group, 14.8%)

4) a subgroup that responded to all ADKS items with high accuracy (general-focused group, 35.6%) (**Figure 1**).

### Univariate analysis of the factors associated with AD literacy

Most participant characteristics varied significantly among the four subgroups, except for gender. The general-focused subgroup was older, more educated, more engaged as general practitioners, had a more extended history in health care, and held higher professional titles (*P* < 0.05) (**Table 1**).

In the community doctors' clinical practice, the four groups significantly differed in their view that elderly patients mainly visit due to physical illness but not their opinions on cognitive impairment or emotional and behavioural issues. There were also significant differences in how often they took no action or conducted cognitive screenings for elderly patients with recent cognitive decline (*P* < 0.01). Yet, the rate of referring patients to higher-level hospitals was similar across all subgroups (**Table 1**).

### Multivariate analysis of the factors associated with AD literacy

Compared to the general-focused group, age was associated with the physical issue-focused group (OR = 0.415), the severity-focused group (OR = 0.954), and the knowledge-uncertainty group (OR = 0.960). General practitioners and community psychiatrists were less likely to be in the severity-focused group (general practitioner: OR = 0.385; community psychiatrist: OR = 0.236) and the physical issue-focused group (general practitioner: OR = 0.415; community psychiatrist: OR = 0.200) and even less so in the knowledge-uncertainty group (general practitioner: OR = 0.247; community psychiatrist: OR = 0.203).

Lower educational backgrounds strongly indicated membership in the knowledge uncertainty group. At the same time, higher professional titles, specifically associate and chief consultants, were less likely to be associated with this group (**Table 2**).

**Table 2:** Multivariable-adjusted odds (and 95% CI) for each subgroup*

Variables	B	SE (B)	Wald x^2^	*P-*value	OR	95% CI
**Severity-focused group**						
Age	−0.047	0.016	8.424	0.004	0.954	0.925–0.985
Medical practitioner type						
*General practitioner*	−0.954	0.302	9.964	0.002	0.385	0.213–0.696
*Psychiatrist*	−1.445	0.404	12.795	0.000	0.236	0.107–0.520
Memory clinic in community health service centre	0.864	0.433	3.977	0.046	2.373	1.015–5.548
Estimating that higher proportion of patients visit community for physical conditions	−0.667	0.169	15.655	0.000	0.513	0.369–0.714
Taking no action for elderly patients with recent cognitive decline	0.589	0.185	10.091	0.001	1.802	1.253–2.592
**Physical issue-focused group**						
Medical practitioner type						
*General practitioner*	−0.879	0.290	9.172	0.002	0.415	0.235–0.733
*Psychiatrist*	−1.611	0.394	16.688	0.000	0.200	0.092–0.433
Time working in clinical or public health						
*5–9 y*	0.674	0.329	4.202	0.040	1.963	1.03–3.740
Time working in community health centre						
*<5 y*	−0.597	0.302	3.911	0.048	0.550	0.305–0.995
Memory clinic in area	−0.628	0.255	6.093	0.014	0.533	0.324–0.879
Estimating that higher proportion of patients visit community for physical conditions	−0.559	0.150	13.842	0.000	0.572	0.426–0.768
Taking no action for elderly patients with recent cognitive decline	0.407	0.170	5.736	0.017	1.503	1.077–2.098
**Knowledge-uncertainty group**						
Age	−0.040	0.017	5.495	0.019	0.960	0.928–0.993
Education						
*Technical secondary school*	1.697	0.633	7.182	0.007	5.456	1.577–18.874
*Junior college*	1.253	0.546	5.260	0.022	3.500	1.200–10.208
Medical practitioner type						
*General practitioner*	−1.400	0.312	20.163	0.000	0.247	0.134–0.454
*Psychiatrist*	−1.596	0.427	13.963	0.000	0.203	0.088–0.468
Professional title						
*Deputy senior professional*	−1.138	0.483	5.551	0.018	0.320	0.124–0.826
*Intermediate professor*	−0.764	0.372	4.211	0.040	0.466	0.224–0.966
Estimating that higher proportion of patients visit community for physical conditions	−0.835	0.201	17.336	0.000	0.434	0.293–0.643
Taking no action for elderly patients with recent cognitive decline	0.454	0.216	4.414	0.036	1.575	1.031–2.406

β – standardised regression coefficient, CI – confidence interval, OR – odds ratio, SE (B) – standard error of B

*The odds values are computed for the association of multiple baseline characteristics and clinical practice with the classification into the severity-focused group, physical issue-focused group, and knowledge uncertainty group compared with the general-focused group.

In clinical practice, community doctors who see physical conditions as the main reason for elderly patient visits tend to be less associated with membership in the severity-focused group (OR = 0.513), the physical issue-focused group (OR = 0.572), and the knowledge uncertainty group (OR = 0.434). Conversely, a lack of support provision for elderly patients with cognitive impairment correlates positively with membership in these groups, with the corresponding ratios indicating a higher probability of association (**Table 2**).

## DISCUSSION

This study presents the AD knowledge architecture among Chinese community doctors. Additionally, it explores the role of demographic characteristics and clinical practice in shaping the diversity of knowledge structures. Our research identified four distinct AD knowledge structures among Chinese community doctors, namely, the severity-focused group, the physical issue-focused group, the knowledge uncertainty group, and the general-focused group. Several characteristics were found to be associated with the knowledge structure, including advanced age, higher educational attainment, engagement as a general practitioner, and extensive experience in health care service, along with the perception that a greater proportion of patients seek care for physical reasons, are associated with a comprehensive understanding of AD knowledge among community doctors. Conversely, community doctors who did not provide treatment measures for patients with recent cognitive impairment tended to have a limited understanding of AD knowledge.

In contrast to previous studies [6,37] that found a correlation between younger age and more excellent knowledge of dementia, our study showed that older individuals were more inclined to have a comprehensive understanding of dementia. This finding aligns with the research conducted by Scerri et al. [10], which demonstrated that advanced students exhibited more excellent knowledge and a more positive attitude. The difference in study populations may explain this discrepancy. A study by Zhao et al. focused on older adults residing in China and Australia [37], while Mat Nuri et al. examined pharmacists, who may experience a decline in their ability to acquire new knowledge as they age and become less familiar with specific areas [6]. In contrast, our study focused on health care professionals and found that their understanding of AD is enhanced by continuous education, involvement in AD-related research and clinical activities, and increased age. Furthermore, the accumulated clinical experience of senior community doctors may facilitate quicker AD identification and diagnosis, bolstering their knowledge in this area. These findings suggest that the influence on dementia knowledge structure is not singular but the result of multiple intertwined factors.

Our study found that general practitioners were more inclined to understand AD knowledge comprehensively. This finding is consistent with a Spanish survey on the AD knowledge levels of community pharmacists and general practitioners which revealed that general practitioners scored higher on diagnosis, treatment, and symptom-related aspects [7]. Similarly, a study by Wang et al. in China showed that general practitioners had greater knowledge levels of AD than individuals in other groups [12]. This may be attributed to the fact that community doctors serve as primary health care providers in China, which affords them more opportunities to interact with AD patients and participate in initial diagnosis and treatment services. Consequently, they are more likely to actively seek information related to dementia to assist them in their clinical work. In community health services, general practitioners prioritise the education of patients and their families regarding AD, improving disease awareness and self-management capabilities. Concurrently, they persist in deepening their understanding of AD.

Higher education levels may also be associated with AD knowledge [10,13,28,37–39]. In our study, individuals in the general-focused group tended to have higher education levels. Previous research has shown that a university degree or higher predicts higher knowledge of life impact, risk factors, and care [39,40]. One possible reason is that higher education-level individuals have greater access to resources to learn relevant knowledge, such as AD-specific training. Another reason could be their more vital ability to engage in international communication, leading to greater motivation for proactive learning – however, Mat Nuri et al. [6] and Kafadar et al. [41] found that ADKS scores were independent of education level, suggesting that the factors influencing AD knowledge are not dependent solely on education but are also related to occupation, age, and other factors. A positive correlation was also found between self-assessed dementia knowledge and participation in specialised dementia education courses [32]. Therefore, establishing dementia education and training programmes to enhance the understanding of primary health care doctors and caregivers who frequently encounter dementia patients can improve their diagnostic and treatment capabilities.

This study is the first to link Chinese community doctors' clinical practice and experience to AD knowledge. The results showed that community doctors who believed that more than 50% of patients sought treatment due to physical conditions were more likely to have a comprehensive understanding of AD knowledge. Conversely, community doctors who did not provide support for elderly patients with recent cognitive impairment had a limited grasp of AD knowledge. Previous studies have also explored the relationship between clinical practice and AD knowledge. Scerri et al. [10] observed a significant correlation between clinical experience and a more positive attitude toward AD diagnosis and treatment. Amado and Brucki's study showed that professional skills positively influenced ADKS scores [28]. A study on the impact of educational experiences on nursing students' knowledge and attitudes toward Alzheimer disease found that experiential learning during clinical internships increased students’ knowledge and improved their attitudes toward AD [11]. These findings indicate the relevance of clinical practice experience to AD knowledge. They suggest community doctors compensate for educational gaps through continuous improvement in their learning system due to practical experience. Future research should include a broader range of clinical practices to improve the integration of learning and training for a better understanding of AD knowledge. This approach will help build a theoretical foundation based on practical experience and refine the theoretical framework through practical application.

This study included participants from various provinces and autonomous regions in China and provided insights into the heterogeneity of AD knowledge among community doctors. The study highlighted the potential heterogeneity in the ADKS knowledge structure among community doctors to offer a unique perspective on the population's mastery of AD knowledge. Our research is the first to use latent class analysis to explore the knowledge structure of AD in a large sample of Chinese community doctors and its relationship with clinical practice. A previous study examined only the latent structure of ADKS items without considering the heterogeneity of AD knowledge among different populations [8]. Furthermore, prior research has only assessed the impact of clinical experience on AD knowledge without clearly defining the specific content of clinical practice [11,28]. Importantly, we provide directions for training interventions for the early diagnosis of AD. Thus, our study offers a scientific basis for promoting a comprehensive knowledge structure through clinical practice and providing a theoretical foundation for improving human resources for the early diagnosis and treatment of AD.

Despite these advantages, the present work still has several limitations. First, while the study sample encompasses community doctors from most Chinese provinces, it needs a further exploration of rural community doctors, impeding a clear delineation of urban-rural disparities in AD knowledge. Second, the questionnaire relied on self-reported measurements and lacked specific information on past clinical practice content. The self-reporting of clinical practices may be subject to recall bias and may differ from actual workplace records. However, robust mechanisms for monitoring cognitive screening practices are currently lacking. Third, information on the training experiences of community doctors in the field of cognitive impairment is complex to collect due to the need for a systematic registration system, resulting in significant variation in self-reported training hours. Finally, due to the unique nature of the Chinese health care system, there may be overlap between the rotation of medical positions and years of clinical or public health work, which could lead to differences in the assessment of the impact of work time on different AD knowledge structures compared to the results of this study.

## CONCLUSIONS

This study explored the different structures of AD knowledge from the perspectives of knowledge accumulation, experience accumulation, and skill development. In subsequent research endeavours, it is imperative to integrate qualitative interviews and focus groups to delve deeper into the underpinnings of varying dementia knowledge structures. Further, we need to broaden the study scope to cover a more diverse sample demographic, particularly community physicians in rural settings, and to uncover regional disparities in dementia knowledge frameworks. Future research should consider other aspects, such as cultural values related to dementia and regional economic conditions when exploring AD knowledge frameworks. Furthermore, it is necessary to establish a comprehensive registration system for training experiences and monitoring mechanisms for specific clinical practice content. Such enhancements will facilitate longitudinal latent class transition analyses and pre- and post-AD knowledge training, thereby evaluating the patterns of knowledge structure evolution in the context of educational interventions and professional development.

## Additional material


Online Supplementary Document
