# Supplementary material for: Heterogeneity in the level of dementia literacy among community doctors in China: A latent profile analysis
Source: J Glob Health. 2024 Nov 15;14:04161. doi: 10.7189/jogh.14.04161 (PMC11565466; doi:10.7189/jogh.14.04161)
Supplement: Online Supplementary Document [file jogh-14-04161-s001.pdf]

Supplemental materials

Table S1. Variables and coding methods

Variable	Coding methods
Sex	1=male; 2=female
Education	1 = technical secondary school; 2 = junior college;<br>3 = bachelor degree; 4 = master degree or above
Medical practitioner type	1 = general practitioner; 2 = psychiatrist;<br>3 = family doctor; 4 = public health doctor
Time working in clinical or public health	1 = less than 5 years; 2 = 5-9 years;<br>3 = 10-14 years; 4= more than 15 years
Time working in community health center	1 = less than 5 years; 2 = 5-9 years;<br>3 = 10-14 years; 4= more than 15 years
Professional title	1=senior professional; 2=deputy senior professional;<br>3=intermediate professor; 4=junior professor; 5=others
Memory clinic in area	1=yes; 2=no
Memory clinic in community health service center	1=yes; 2=no

**Clinical practice**	
Q1. The estimates of the proportion of reasons that elderly patients visit community doctors and seek advice	
Physical conditions	1= ≥ 50%; 2= ≤ 49%
Cognitive decline	1= ≥ 50%; 2= ≤ 49%
Emotional and behavioral problems	1= ≥ 50%; 2= ≤ 49%
Q2. Proactively inquiring about changes in cognitive functions for elderly patients presenting with emotional and behavioral problems or physical conditions	1= usually; 2= less often or none
Q3. The estimates of the frequency of clinical management for elderly patients with recent cognitive decline	
Taking no action	1= usually; 2= less often or none
Conducting cognitive screenings	1= usually; 2= less often or none
Referring to higher-level hospitals based on the outcomes of cognitive screening	1= usually; 2= less often or none
Making direct referrals to higher-level hospitals	1=yes; 2=no

Table S2. Potential latent analysis fitting indicators for the total ADKS scores of community doctors (n=1,288)

Models	AIC	BIC	aBIC	Entropy	VLMR-LRT	BLRT	Category probability
1-cluster	40472.86	40627.69	40532.39	-	-	-	-
2-cluster	36920.63	37235.44	37041.68	0.942	<0.001	<0.001	0.72/0.28
3-cluster	36066.55	36541.35	36249.11	0.844	<0.001	<0.001	0.50/0.27/0.23
4-cluster	35672.83	36307.63	35916.91	0.814	<0.001	<0.001	0.35/0.29/0.21/0.15
5-cluster	35526.23	36321.00	35831.82	0.825	0.187	<0.001	0.25/0.33/0.21/0.02/0.19
6-cluster	35372.26	36327.02	35739.37	0.823	0.146	<0.001	0.19/0.26/0.08/0.01/0.25/0.21
